# Emerging mechanisms and promising approaches in pancreatic cancer metabolism

**DOI:** 10.1038/s41419-024-06930-0

**Published:** 2024-08-01

**Authors:** Hao Wu, Mengdi Fu, Mengwei Wu, Zhen Cao, Qiyao Zhang, Ziwen Liu

**Affiliations:** 1 Department of General Surgery, Peking Union Medical College Hospital, Chinese Academy of Medical Sciences and Peking Union Medical College, Beijing, 100730 China; 2 Department of Clinical Medicine, Peking Union Medical College Hospital, Chinese Academy of Medical Sciences and Peking Union Medical College, Beijing, 100730 China

**Keywords:** Cancer metabolism, Pancreatic cancer

## Abstract

Pancreatic cancer is an aggressive cancer with a poor prognosis. Metabolic abnormalities are one of the hallmarks of pancreatic cancer, and pancreatic cancer cells can adapt to biosynthesis, energy intake, and redox needs through metabolic reprogramming to tolerate nutrient deficiency and hypoxic microenvironments. Pancreatic cancer cells can use glucose, amino acids, and lipids as energy to maintain malignant growth. Moreover, they also metabolically interact with cells in the tumour microenvironment to change cell fate, promote tumour progression, and even affect immune responses. Importantly, metabolic changes at the body level deserve more attention. Basic research and clinical trials based on targeted metabolic therapy or in combination with other treatments are in full swing. A more comprehensive and in-depth understanding of the metabolic regulation of pancreatic cancer cells will not only enrich the understanding of the mechanisms of disease progression but also provide inspiration for new diagnostic and therapeutic approaches.

## Key facts

- Metabolic reprogramming is one of the key hallmarks in pancreatic cancer.
- Targeting key enzymes in metabolic pathways can affect the progression of pancreatic cancer.
- The complex tumor microenvironment of pancreatic cancer creates metabolic heterogeneity.
- Clinical trials exploring metabolic-based treatments for pancreatic cancer have been initiated.
- Advanced technologies (including preclinical models and detection technologies) continue to promote progress in the field.

## Key questions

- Influencing redox homeostasis brings hope for cancer treatment.
- Polyamine metabolism and microbial metabolic targets still need further exploration.
- Interactions between metabolism and epigenetics and metabolic cell death are blue oceans.
- Systemic metabolism can also serve as a potential pathogenic mechanism and therapeutic target.
- Breakthroughs are urgently needed to overcome the low specificity and side effects of metabolic therapy.

## Introduction

Tumour cells have many unique characteristics, and many of these characteristics, are directly controlled by cell metabolism or amenable to regulation by specific metabolites [1]. Since the Warburg effect was identified a century ago [2], a variety of metabolic reprogramming events have been revealed in tumour cells [3]. The Warburg effect refers to persistent glycolysis even when oxygen is abundant, which provides ATP but is primarily a precursors for the synthesis of various biological macromolecules [4]. With the rapid development of new technologies, it has been gradually discovered that… tumour metabolism involves an overall change at multiple levels of the metabolic network and plays an important role in promoting the occurrence and development of tumours [5, 6].

Metabolic reprogramming [7], which refers to the adaptive changes in the balance of anabolism and catabolism that occur in tumour cells during the malignant development process to meet the large demand for materials and energy, has gradually become recognized as one of the crucial hallmarks of cancer [8]. This reprogramming allows tumour cells to gain advantages and survive in the nutrient-starved tumour microenvironment (TME) caused by rapid proliferation [9].

In contrast to the booming research field, only a few metabolism-based cancer drugs have been successfully developed and are still in the nascent stage due to their low specificity and undesirable side effects [10]. These strategies targeting the intrinsic metabolism of cancer cells usually do not consider the complex crosstalk of noncancerous stromal cells and immune cells, still awaiting further research [11–13].

Pancreatic cancer (PC), is an aggressive cancer with a poor prognosis [14]. The overall five-year survival rate is less than 13%, and it is expected that PC will rank second in malignant tumour-related deaths in the United States by 2030 [15]. Exploring the metabolic regulatory network and underlying mechanisms, formulating comprehensive treatment strategies, and promoting precise treatment based on individual patients are expected to improve the overall prognosis.

**Fig. 1:**
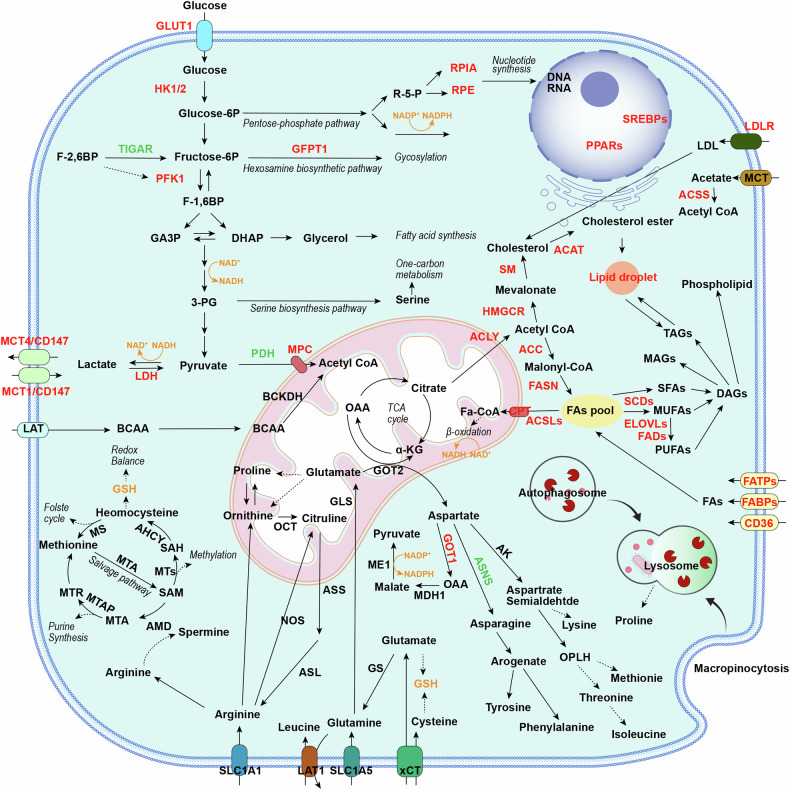
Integrated altered metabolic pathways in pancreatic cancer cells. Changes in key enzymes and transporters lead to metabolic reprogramming of glucose, amino acids, lipids, etc., and meet the large demand for materials and energy during the malignant proliferation of tumor cells. Enzymes and transporters shown in red indicate significant overexpression; enzymes and transporters shown in green indicate significant downexpression; enzymes shown in black indicate no significant changes or unknown; metabolites shown in orange indicate that they were involved in redox balance; solid arrows imply shifts or bioconversions; dashed lines indicate that the reaction is not direct; italics indicate metabolic pathways or processes. 3-PG 3-bisphosphoglycerate, ACC acetyl-CoA carboxylase, ACAT acetyl-CoA acetyltransferase, ACLY ATP citrate lyase, ACSS acyl-CoA synthetase short-chain family, AHCY adenosylhomocysteinase, ASNS asparagine synthetase, ASL argininosuccinate lythase, ASS argininosuccinate synthase, AK aspartic kinase, AMD aAdenosylmethionine decarboxylase, BCAA branched-chain amino acids, BCKA branched alpha-ketoacids, BCKDH branched alphaketoate dehydrogenase, CE cholesterol ester, CS citrate synthetase, DAG diacylglycerol, DHAP: dihydroxyacetone phosphate, ELOVL FA elongase, FA fatty acid, FABP fatty acid-binding protein, FADS FA desaturase, FASN fatty acid synthase, FATP: fatty acid transport protein, Fructose-6P fructose 6-phosphate, F- 1:6BP fructose 1:6-bisphosphate, F-2:6BP fructose 2:6-bisphosphate, GA3P glyceraldehyde 3-phosphate, GLUT1 glucose transporter 1, GSH glutathione synthesis, GLS glutaminase, GFPT1 glutamine:fructose 6-phosphate amidotransferase 1, Glucose-6P glucose 6- phosphate, GOT1 cytoplasmic aspartate Transaminase, GOT2 mitochondrial aspartate transaminase, HK1/2 hexokinase 1/2, HMGCR: 3-hydroxy-3-methylglutaryl coenzyme A reductase, HMG-CoA 3-hydroxy-3-methylglutaryl coenzyme A, ME1 malic enzyme, LDLR low-density lipoprotein receptor, LDH lactate dehydrogenase, MAG monoacylglycerol, MCT monocarboxylate transporter, MDH1 malate dehydrogenase 1, MTA 5:-methylthioadenosine, MTAP 5-methylthioadenosine, MS methionine synthase, MUFA monounsaturated fatty acid, NAD nicotinamide adenine dinucleotide, NADPH nicotinamide adenine dinucleotide phosphate, NOS nitric oxide synthase, OCT ornithine transcarbamylase, OPLH o-phospho-l-homoserine, PDH pyruvate dehydrogenase, PFK1: phosphofructokinase 1, PRODH1 proline oxidase, PUFA polyunsaturated fatty acid, R-5-P ibose-5-phosphate, RPE ribulose-5-phosphate epimerase, RPIA ribose-5-phoshate isomerase, SAH S-homocysteine; SAM S-adenosine methionine, SCD stearoyl-CoA desaturase, SFA saturated fatty acid, SM squalene monooxygenase, TAG triacylglycerol, TCA tricarboxylic acid.

This review summarizes the main themes that are currently under investigation in the context of tumour metabolism and provides an overview of the current status of basic research and clinical trials targeting key enzymes and signaling pathways in PC metabolism (Fig. 1).

## Metabolic reprogramming of tumour cells

### Nucleotide metabolism

The enhanced synthesis and use of nucleotide triphosphates (NTPs) is a universal metabolic dependence across different cancer types [16]. Oncogenic drivers increase nucleotide biosynthetic capacity, which is a prerequisite for cancer initiation and progression [17]. The clinical utility of nucleotide synthesis inhibitors, the first antineoplastic agents discovered, has been demonstrated in many cancers [18]. As they have been extensively studied and due to space limitations, we summarize the key enzymes and inhibitors in nucleotide metabolism in Text Box S1.

In addition to the de novo and salvage pathways, pancreatic ductal adenocarcinoma (PDAC) cells can also acquire nucleotides through autophagy, specifically through the degradation of cellular nucleic acids in autophagosomes, especially in hypoxic and nutrient-poor regions of the TME [19]. Recent studies have also shown that nucleotide metabolism is not only a therapeutic target for chemotherapy but also has the potential to become a sensitization target for immunotherapy, which we will describe later [20].

### Glycolysis and OXPHOS

Otto Warburg first discovered in the late 1920s that increased aerobic glycolysis in tumour cells [21], also known as the Warburg effect, allows tumour cells to survive under the harsh conditions of cancer progression [22]. Glycolysis is the primary way PC cells generate energy to sustain malignant proliferation, including under hypoxic conditions [23, 24]. Work in mouse models also demonstrated that glycolysis is the major metabolic effector of oncogenic KRAS [25].

First, the expression of glucose transporter 1 (GLUT1) is upregulated, increasing glucose uptake to enhance subsequent aerobic glycolysis [26]. LIMS1 enhances GLUT1 expression and membrane translocation, which promotes cancer cell survival under oxygen-glucose deprivation [27]. Key enzymes involved in glycolysis play a role in PC. Hexokinase 1/2 (HK1/2) converts glucose into glucose-6-phosphate (G-6-P), the rate-limiting first step in glycolysis. The binding of HK2 to mitochondria increases the glycolytic capacity and promotes PDAC immortalization [28]. Recent research has shown that the endogenous cellular metabolic checkpoint STING can limit aerobic glycolysis by targeting HK2 [29]. Phosphofructokinase (PFK) catalyses the second critical step in glycolysis [30]. The production of the PFKFB-4 and PFKFB-3 isoenzymes is induced by hypoxia in PC [31]. Pyruvate kinase (PK) catalyses the conversion of phosphoenolpyruvate to pyruvate, which is the final rate-limiting step of glycolysis and can transfer metabolites to branch pathways [32]. Some studies have shown that methionine can oxidatively activate PKM2 to promote PC metastasis [33], while other studies have shown that PKM2 expression is not required for carcinogenesis or progression in PDAC [34]. Under conditions of glucose deficiency, fructose can be taken up and metabolized by PDAC cells to promote adaptive survival [35].

The expression of several enzymes involved in glycolysis, such as aldolase [36], glyceraldehyde 3-phosphate dehydrogenase (GAPDH) [37], phosphoglycerate kinase (PGK) [38], phosphoglycerate mutase (PGAM) [39], and alpha enolase (ENO-1) [40], is upregulated in PC. Lactate dehydrogenase (LDH) is overexpressed in PDAC [41], and LDH-mediated catalysis of pyruvate to lactate is significantly increased [42]; thus, LDH may be an effective therapeutic target [43].

In contrast to glycolysis, phosphorylated pyruvate dehydrogenase kinase 1 (PDHK1) inhibits oxidative phosphorylation (OXPHOS) in PDAC [26]. Nonetheless, some studies have shown that OXPHOS is not always suppressed; rather, it can be reactivated under certain conditions [44]. The oncogene KRAS and the loss of LKB1 may drive the upregulation of OXPHOS in cancer [44]. The transcription factor ISL2 can regulate the expression of metabolic genes and affect OXPHOS [45]. As tumours proliferate rapidly, cells in the tumour core become hypoxic, and OXPHOS decreases. Several studies have demonstrated that OXPHOS inhibitors can serve as promising therapeutic agents for treating PDAC [46]. The molecular mechanism underlying the relationship between OXPHOS and PDAC progression still requires in-depth study with bright prospects (Text Box S2).

### Amino acid metabolism

Amino acid metabolism, which can affect cancer cell status and systemic metabolism through energy metabolism and signal transduction, is deregulated in PDAC [47].

Glutamine is the most abundant nonessential amino acid in the human body [48]. Cancer cells can convert glutamine into lactic acid, participate in a glucose-free metabolic pathway, take up glutamine, and promote the synthesis of nonessential amino acids and nucleotides through carbon or nitrogen metabolism [49]. Generally, glutamine can be converted in the mitochondria to supplement the tricarboxylic acid (TCA) cycle, or it can be completely oxidized to produce ATP. Many studies have confirmed that glutamine plays a very important role in the migration and invasion of PDAC cells [50–52] and relies on noncanonical metabolic pathways. Glutamine-derived aspartate is converted into oxaloacetate by aspartate transaminase GOT1; subsequently, oxaloacetate is converted into malate and then pyruvate, which can potentially maintain redox homeostasis [53]. Loss of SIRT5 promotes tumorigenesis by increasing noncanonical glutamine utilization through GOT1 and is expected to serve as a suppressor in PDAC [54]. GOT1 inhibition-mediated impairment of redox balance synergizes with radiotherapy mouse models [55]. The adaptation of PDAC cells to nutrient deprivation is reversible, and glutamine synthetase (GS) is expected to become a therapeutic target for patients with PDAC [56]. Alanine, serine, and cysteine transporter 2 (ASCT2) is responsible for glutamine transport [57]. Recent studies have shown that CD9 promotes the plasma membrane localization of the glutamine transporter ASCT2, thereby enhancing glutamine uptake [58]. In preclinical models, PI3K-C2γ deficiency can overactivate the mTORC1 pathway and reprogram glutamine metabolism, increasing invasiveness [59]. Targeting glutamine metabolism may provide therapeutic avenues for treating PDAC [60], and glutamine mimicry inhibits tumour progression through asparagine metabolism [61].

In addition, many studies have found that other amino acids, such as serine, tryptophan, methionine, and branched-chain amino acids (BCAAs) such as leucine, play a role in different stages of tumors and are expected to develop new intervention strategies. We summarize them in Text Box S3.

Recently, polyamine metabolism has been redefined as an anticancer strategy and may be involved in antitumour immune responses [62, 63]. The RAS-RAF-MEK-ERK signalling pathway has been shown to control multiple aspects of polyamine metabolism in preclinical models [64]. Arginine deprivation can inhibit the migration, invasion and EMT of PDAC cells [65]. Arginine is the precursor of polyamines, and Lee et al. reported that PDAC cells can synthesize polyamines from glutamine, revealing a new pathway [66]. PDAC can synthesize ornithine from glutamine and support polyamine synthesis through ornithine aminotransferase (OAT), thereby promoting tumour growth [67]. Cancer cells require elevated levels of polyamines to sustain proliferation [67]; microbiota- and diet-related polyamine metabolism will be described later.

### Lipid metabolism

Lipids not only provide energy but are also widely distributed in organelles and serve as second messengers to transduce signals within cells [68]. Therefore, lipid metabolism is increasingly recognized as an important pathway for cancer cells [69, 70]. Lipid metabolism reprogramming can be observed even outside histological tumour boundaries [71], and lipidomic analysis of serum holds promise for detecting PDAC [72].

Exogenous fatty acid (FA) uptake confers metabolic flexibility to cancer cells [73]. Cancer cells can absorb fatty acids through fatty acid transporter proteins (FATPs) [74], fatty acid translocase (CD36) [75], and fatty acid binding proteins (FABPs) [76]. CD36 is thought to be involved in the initiation of metastasis [77] and the promotion of fatty acid β-oxidation to support proliferation [78]. Excess exogenous FAs are stored in lipid droplets (LDs), which sequester FAs in the form of triacylglycerides (TAGs) and sterol esters and can be used for energy production or phospholipid synthesis [79]. Increased LD abundance is related to tumour aggressiveness, and reduced LD abundance can reduce the invasive ability of KRAS-mutant PDAC [80]. β-oxidation of stored lipids can produce acetyl-CoA, which is subsequently shuttled through the TCA cycle, providing a valuable source of ATP and NADPH under conditions of metabolic stress [81].

The de novo synthesis of lipids is a metabolic source for tumour cell growth, even in the presence of exogenous lipids [70]. Acetyl-CoA is the main substrate for lipid synthesis. Cancer cells can upregulate acetyl-CoA synthetase 2 (ACSS2) expression to generate acetyl-CoA from acetate [82] or upregulate ATP-citrate lyase (ACLY) expression to convert citrate to acetyl-CoA [83]. Glucose undergoes pyruvate oxidation through the TCA cycle, while glutamine promotes citric acid production through reductive carboxylation [84].

The regulation of de novo lipogenesis mainly occurs at the transcription level, and sterol regulatory element binding proteins (SREBPs) can regulate genes related to fatty acid and cholesterol synthesis and uptake [85]. Liver X receptors (LXRs) [86], whose inhibitory ligands can hinder cell proliferation in various forms of cancer, regulate FA and cholesterol metabolism [87]. Acetyl-CoA carboxylase (ACC) [88], fatty acid synthase (FASN) [89], and stearoyl-CoA desaturase (SCD1) [90] are rate-limiting enzymes for FA synthesis and are significantly highly expressed in PDAC. ACC1 is genetically regulated by SREBP at the transcription level [91]. In recent years, FASN has received increasing attention as a potential target for cancer therapy, and its high expression is associated with poor survival and gemcitabine resistance [92]. Under hypoxic conditions, the flux from glucose to acetyl-CoA is reduced, and saturated fatty acids are regulated by SCD1, reducing the conversion to monounsaturated fatty acids [93]. SCD1 expression is associated with poor prognosis in PDAC patients and can protect cancer cells from ferroptosis, indicating its antitumour potential [94].

Cholesterol plays a key role in maintaining membrane integrity and fluidity and regulating cell signalling events [95]. Abnormal cholesterol metabolism can support PDAC growth [96]. The uptake of cholesterol depends on the LDLR-mediated endocytosis pathway. Low-density lipoprotein (LDL) particles bind to the LDL receptor (LDLR) and are eventually internalized and then reach the lysosome to release free cholesterol [97]. Decreased total cholesterol and LDL levels may be associated with the development of PDAC [98]. Some studies have also shown that LDLR expression is positively correlated with poor prognosis and recurrence [99]. Cholesterol is synthesized through the mevalonate pathway and is regulated by several key enzymes, such as acetyl-CoA acetyltransferase (ACAT) [100], 3-hydroxy-3-methyl-glutaryl-CoA reductase (HMGCR) [101], squalene monooxygenase (SM) [102], and sterol-O-acyltransferase (SOAT) [103], which may provide new ideas for the treatment of PDAC [104]. In contrast, the level of high-density lipoprotein (HDL)-cholesterol, which is associated with cholesterol efflux, is significantly inversely related to the risk of cancer [105]. In preclinical models, disruption of cholesterol biosynthesis promotes a shift to a basal phenotype, conferring a poor prognosis [106]. Targeting SOAT1 can affect p53 mutant PDAC organoids that are sensitive to cholesterol metabolism and impair tumor progression [103].

Fatty acid oxidation (FAO), also known as β-oxidation, is increased in many cancer cells because cancer cells can use fatty acid (FA) catabolism to proliferate when ATP is depleted [107, 108]. FAO can participate in tumorigenesis [109] and tumour metastasis [110] and may be related to cachexia [111]. Among different fatty acids, polyunsaturated fatty acids (PUFAs) are easily oxidized, with lipid peroxidation leading to various types of cell death, while saturated fatty acids (SFAs) and monounsaturated fatty acids (MUFAs), on the contrary, are thought to promote cancer growth [112, 113].

Finally, the sophisticated mechanisms and functions of phospholipid metabolism [114] and sphingolipid metabolism [115] have also been increasingly revealed. Increased glycosphingolipid biosynthesis can localize KRAS to the plasma membrane [116]. MUFAs and their linked ether phospholipids play a key role in maintaining reactive oxygen species (ROS) homeostasis [117].

### Redox homeostasis

Cellular redox homeostasis is an important process for cell survival, and ROS can cause damage to cellular components [118]. In addition, cancer cells have an increased demand for nicotinamide adenine dinucleotide (NAD+), which leads to a restorative stress state [119]. Several transcription factors are activated by ROS, regulate the redox status of cells and are implicated in carcinogenesis [120]. New technologies for measuring and manipulating reducing stress offer promise for cancer treatment [121].

As mentioned previously, the glutamine metabolic pathway in PDAC cells is often referred to as the noncanonical pathway. Glutamine deprivation [122] and the inhibition of GOT1 [123] or GOT2 [124, 125] increase intracellular ROS. CRISPR/Cas9 ablation of Glutamate–Ammonia Ligase (GLUL) in PDAC mouse models reduced tumor growth [51]. Cancer cells rely on cysteine-derived metabolites such as glutathione (GSH) and CoA to alleviate ROS [118, 126]. GSH is the most abundant antioxidant in cells mainly to scavenge free radicals and maintain cellular redox homeostasis [127]. Cysteine is taken up by SLC7A11 (also called xCT) [128]. The expression of SLC7A11 and cystine uptake can be mediated by mitochondrial calcium uniporter (MCU), potentially inhibiting PDAC metastasis [129].

Ferroptosis is caused by the excessive accumulation of lipid ROS [130]. The Fenton reaction produced by labile iron in cancer cells promotes the peroxidation of membrane-bound lipids containing polyunsaturated fatty acids [131]. In preclinical models, cysteine depletion and promotion of ferroptosis inhibited PDAC growth [132]. NRF2 can activate downstream suppressor genes through transcription, the trans-sulfur pathway can convert methionine into cysteine, and the mTOR pathway can increase GPX4 protein synthesis, all of which can improve ferroptosis resistance [133]. Cysteine depletion can induce ferroptosis [132]. ROS-enhanced phototherapy via the Nrf2-mediated stress-defence pathway and ferroptosis can inhibit PDAC [134]. Increasing the labile iron in cancer cells can induce ferroptosis, while iron accumulation can also promote tumour progression [135, 136]. Recent groundbreaking studies expand our understanding of the mechanisms of 7-Dehydrocholesterol (7-DHC) in ferroptosis through cholesterol metabolism [137, 138]. The unique ferroptosis response mediated by PHLDA2 is likely independent of ACSL4 and does not require common ferroptosis inducers [139].

Several other metabolic targets that modulate ROS toxicity are also being studied preclinically [140, 141]. Inhibiting nicotinamide phosphoribosyl transferase (NAMPT) to block NAD+ synthesis can inhibit tumour growth [142] and has been carried out in clinical trials [143, 144]. New concepts and attempts related to NAD+ metabolism are being made, but its double-edged sword effect reflects the complexity of regulating redox homeostasis in cancer [145, 146].

### Other metabolic alterations

There are also several metabolism-related targets or pathways that have not been covered in previous chapters. KRAS mutations are among the earliest events in pancreatic carcinogenesis and can drive common metabolic programs and promote tumour progression [147]. In the normal pancreas, acinar cells use amino acids to synthesize digestive enzymes, and ductal cells are mainly responsible for the transport of peptides and hormones. In the case of KRAS mutations, acinar cells can transform into ductal cells and are considered the origin of PDAC [148]. These changes also occur in ductal cells [149]. In the transformed state, KRAS PDAC cells possess metabolic programs intrinsic to acinar and ductal cells [150].

To adapt to harsh environments, PDAC cells rely on lysosomes for nutrient degradation and regeneration, such as through macropinocytosis [151] and autophagy [152], to obtain sufficient fuel for survival [153, 154]. Macropinosomes can nonselectively internalize and absorb a large amount of extracellular fluid and ultimately metabolize amino acids into central carbon to support the growth of cancer cells [155]. Oncogenic RAS induces macropinocytosis [156] and is regulated by EGFR-Pak signalling [157] and syndecan 1 (SDC1) [158]. Autophagy can degrade cellular macromolecules and organelles, maintain cell homeostasis and survival [159], and maintain PDAC growth [160]. A lack of extracellular supplies enhances autophagy [161], and increased autophagy on ferritin maintains iron availability and thereby promotes tumour progression [162]. Research has shown that autophagy promotes immune evasion in PDAC by degrading MHC-I [163] and that the combination of MEK and autophagy inhibition may inhibit PDAC [164]. Preclinical model results further support the critical role of autophagy in tumor maintenance [160].

Because metabolic reprogramming causes differences in metabolites in cells, epigenetic modifications such as lactylation [165], succinylation [166], glycosylation [167] and SUMOylation [168] may also cause different trends in tumour development. For example, SIRT4 can inhibit tumorigenesis by inducing autophagy [169], while Smarcd3 can establish aggressive lipid metabolism remodelling [170]. The interaction between metabolic reprogramming and epigenetics has gradually become the focus of recent research [171–173]. Moreover, some inhibitors have been tested in preclinical models [174].

Recent studies have discovered new forms of regulated cell death resulting from imbalances in cellular metabolism [175]. Copper-dependent signalling pathways were also identified and characterized [176]. Lysosomal function is upregulated in cancer cells and TRPML1 is a cation channel with dual permeability to Ca^2+^ and Zn^2+^, but excessive Zn^2+^ will hinder mitochondrial function and eventually lead to cell death [177]. Disulfide death relies on SLC7A11-mediated cystine transport, which may be resistant to ferroptosis therapy, but is glucose and NADPH dependent [178]. Disruption of intracellular pH balance, such as the selective inhibitor JTC801, triggers alkaline death by blocking OPRL1 and inhibits PDAC growth [179]. Although the role of metabolic cell death is controversial, these may provide insights for novel therapeutic interventions.

## Metabolism of the tumour microenvironment

The PDAC microenvironment is highly intricate and heterogeneous. In addition to cancer cells, there is also an extracellular matrix (ECM), stromal cells, immune cells, etc., and their metabolic interactions play a key role in the occurrence and development of PDAC [180].

PDAC cells are surrounded by dense proliferating connective tissue, which is composed of collagen mesh, leading to hypoxia and nutrient deficiency in tumours [181]. In this environment, cancer cells can activate the PI3K/AKT signalling pathway to enhance glycolysis [182] and even use collagen to provide energy [183]. Connexin-43 channels can transport excess lactate produced by glycolysis and maintain a suitable chemical environment [184]. The lactate receptor GPR81 has also been reported to regulate the lactate transport mechanism [185]. Correspondingly, lactic acid also promotes tumour invasiveness through angiogenesis, immune evasion, and cell migration [186].

Pancreatic stellate cells (PSCs), especially activated PSCs (aPSCs) and cancer-associated fibroblasts (CAFs), are the main components of the PDAC stroma [187, 188]. Inflammatory CAFs can promote PDAC progression [189]. Pancreatic CAFs can support the cellular metabolism of PDAC cells in vitro and in vivo by secreting metabolites [190], which is known as the reverse Warburg effect [191]. Metabolic crosstalk between PDAC cells and CAFs can be achieved by alanine uptake by specific transporters, such as the neutral amino acid transporter SLC38A2 [192]. Lau et al. constructed an organoid-fibroblast co-culture system and found that PDAC cells showed increased pyruvate carboxylation [193]. Cancer cells release tryptophan-derived formate, which can be used by PSCs to support purine nucleotide synthesis [194]. CAFs can secrete lipids such as lysophosphatidylcholine (LPA) and promote PDAC cell progression and AKT activation through the autotaxin-LPA axis [195]. Meanwhile, CAFs can secrete pyrimidines and deoxycytidine, inhibiting the effects of gemcitabine [196], and secrete cytokines and chemokines that support PDAC progression [197]. Recent studies have shown that CAFs can provide bioavailable iron to resist autophagy [198] and secrete cysteine to support glutathione synthesis [199], which induces ferroptosis resistance. Metabolic interactions between cancer cells and PSCs also overcome the redox limitations of cell proliferation [200]. Single-cell sequencing revealed a novel subpopulation of CAFs (named mediated CAFs (meCAFs)) with abnormal glucose metabolism that are associated with a poor prognosis but may better respond to immunotherapy [201]. Because fibroblasts have multiple functions that support or suppress PDAC, it is of great significance to understand the mechanisms and develop targeted therapies.

Nerve invasion is a characteristic of PDAC and is related to prognosis [202]. Neurotrophic factors or neurotransmitters secreted by neurons have been shown to have tumour-promoting effects [203], and the secretion of serine is a newly discovered metabolic crosstalk mechanism [204].

Adipocytes can meet high nutritional demands by secreting adipokines and disrupting lipid metabolism and, in addition, have the potential to transdifferentiate into fibroblast-like cells [205]. Adipocytes may also contribute to the malignant progression of PDAC through metabolic interactions [206], such as through glutamine secretion [207] or through interactions with tumour-associated neutrophils [208].

Immune cells are important components of the TME, and continuously activated inflammatory pathways can promote the occurrence of cancer [13]. The known metabolic patterns of immune cells are mainly different: activated immune cell metabolism resembles the Warburg effect, without obvious OXPHOS; glycolysis in effector T cells increases; and resting immune cells mainly use the tricarboxylic acid cycle and OXPHOS [13].

Macrophages are the most abundant cell type in the TME and play a crucial role in immunity and metabolism [209]. The infiltration and activation of tumour-associated macrophages (TAMs) can promote immune evasion and matrix remodelling in PDAC [210]. The metabolic reprogramming of TAMs through collagen turnover can result in a profibrotic profile [211]. The simultaneous exposure to hyperglycaemia and macrophages can increase the migratory potential of PDAC cells through the epithelial-to-mesenchymal transition (EMT) [212]. TAMs can differentiate into M1 or M2 phenotypes to adapt to the microenvironment [213]; for example, lactate promotes the proto-oncogenic M2-like polarization of TAMs, while through metabolic normalization, TAMs can transform into an anticancer M1 phenotype in preclinical models [214, 215]. Conversely, TAMs can also enhance glycolysis in PDAC cells by secreting cytokines, such as IL-8 [216]. Arginase 1 in immunosuppressive macrophages consumes arginine and inhibits T-cell infiltration [217]. Reducing the levels of the immune metabolite itaconic acid through ACOD1 depletion enhances CAR-macrophage function [218].

T cells are the pioneers of adaptive immune responses, and the infiltration of different types of T cell subsets has different effects on tumours [219]. Single-cell sequencing enables the analysis of the metabolic status and metabolite dynamics of T-cell subsets and their interactions with immunity [220]. Type II cytokines secreted by Th2 cells can stimulate cancer cell-intrinsic MYC transcriptional upregulation to drive glycolysis, accelerating tumour growth [221]. Tryptophan and arginine are required for effector function and T-cell survival [222]. IDO is overexpressed in PDAC and inhibits antitumour T cell responses, and it catalyses the conversion of tryptophan to kynurenine [223]. To meet metabolic demands upon glucose deprivation, tumour-infiltrating lymphocytes (TILs) may be forced to enhance OXPHOS, thereby increasing ROS levels and ultimately leading to dysfunction and failure [224]. Regulatory T cells (Tregs) and myeloid-derived suppressor cells (MDSCs) are also affected by metabolic reprogramming in the TME, which results in immunosuppression [225]. B cells can induce tumour-promoting and immunosuppressive effects. Tumour-associated neutrophils (TANs) can promote a hypoxic microenvironment and immunosuppression [226]. BHLHE40-driven TANs exhibit hyperactivated glycolysis with pro-oncogenic and immunosuppressive functions [227]. However, current research is limited to the main metabolic pathways involved. The roles of other metabolic pathways, such as cholesterol metabolism, and some immune cells, such as natural killer (NK) and CD4+ T cells, are still unclear, and the role of metabolic reprogramming in immune cells remains to be explored. NK cells can exert cytotoxic effects without antigen pre-sensitization, but some metabolic features in the TME hinder NK cell immunotherapy [228]. NK cell dysfunction may be induced by lactate accumulation [229] and competitively inhibited by the active consumption of vitamin B6 in PDAC [230].

Recently, the link between the microbiome and PDAC has attracted considerable interest [231, 232] and may be useful for PDAC detection [233, 234]. Several microorganisms that act on the adenosine pathway have been found to enhance the efficacy of immune checkpoint blockade (ICB) [235]. The expression of genes involved in the polyamine and nucleotide biosynthetic pathways, which are strongly correlated with host tumorigenesis and can serve as predictive markers for the early detection of PDAC, has been shown to be significantly elevated [236]. Recent research has suggested that the microbiome-derived metabolite trimethylamine N-oxide (TMAO) may be a driver of antitumour immunity [237] and that microbiota-derived 3-IAA can influence chemotherapy efficacy [238]. The aryl hydrocarbon receptor in TAMs can be activated by tryptophan-derived microbial metabolites to suppress antitumour immunity [239]. The role of intratumoural bacteria in PDAC requires further study due to their ability to modulate the host immune system and metabolize drugs [240, 241].

The metabolic regulatory role of small extracellular vesicles (sEVs), such as exosomes, as intercellular communication mediators in tumours should not be ignored [242, 243]. Pancreatic cancer-derived extracellular vesicles may influence lipolysis to induce cancer-related cachexia [244]. The detection of sEVs based on lectin-glycan interactions has potential as an early diagnostic marker [245]. Exosomes derived from the tumour microenvironment can also mediate cancer cell metabolism [246].

## Metabolism of the body

In addition to local metabolic reprogramming in tumours, systemic metabolism can also serve as a potential pathogenic mechanism and therapeutic target [247]. A large-scale database study showed that obesity is associated with various cancers [248]. Obesity and diabetes are increasingly recognized as risk factors for PDAC, not only because of the remodelling of the tumour microenvironment but also because of their effects on systemic metabolism [249, 250].

Metabolic syndrome (MetS), a pathological condition characterized by abdominal obesity, insulin resistance, hypertension, and hyperlipidaemia, is associated with the risk of PDAC [251]. In the setting of diet-induced hyperinsulinaemia and obesity, insulin dose-dependently increases the formation of acinar to ductal metaplasia via trypsin and the insulin receptor (InsR) [252]. Stress adaptation through phase-separated organelle stress granules (SGs) mediates the development of PDAC, and obesity may be a driving force behind this process [253]. At the same time, obesity and diabetes are also important factors leading to chemotherapy resistance [254]. Studies have shown that metformin increases the sensitivity of PDAC cells to gemcitabine [255], suggesting the development of new therapies [256]. Clinical trials using related drugs for the treatment of PDAC have been carried out and will be listed later.

There are already many forms and mechanisms of dietary intervention for cancer [257]. However, there is still a long way to go before clinical application. We list several dietary intervention models in Text Box S4.

In addition to diet, physical exercise may reduce the progression of cancer [258]. Moreover, mild cold exposure activates brown fat and hinders glycolysis-based metabolism in cancer cells [259]. Patients with PDAC often experience weight loss and skeletal muscle wasting [260]. Aerobic exercise can promote immune mobilization and exert antitumour effects through the IL-15/IL-15Rα axis [261]. However, due to individual differences in body constitution, caution must be used when applying dietary intervention and physical exercise in cancer treatment.

## Cooperation with other antitumour therapies

### Radiotherapy

Although there is some controversy regarding the survival benefit of this technique, radiation therapy has been used to treat borderline resectable PDAC, providing the possibility of resection [262]. Additionally, metabolic changes in PDAC can cause radio-resistance [263].

Overexpressed MUC1 enhances nucleotide metabolism, facilitates radiation resistance, and is targeted effectively through glycolytic inhibition [264]. Multiple cholesterol synthesis-related genes are associated with radio-resistance in cell lines [265]. Serine and glycine starvation can inhibit the antioxidant response, nucleotide synthesis, and the TCA cycle and has the potential to become a cancer radio-sensitization strategy [266].

### Chemotherapy

Chemotherapy is the most commonly used systemic treatment. Gemcitabine is a type of nucleoside analogue that can interfere with DNA synthesis and block the cell cycle [267].

Gemcitabine-resistant cell lines exhibit increased aerobic glycolysis and reduced ROS levels [268, 269]. Organoid-based metabolomic analysis revealed that PDAC with high glucose metabolism levels is more resistant to chemotherapy and that GLUT1/ALDOB/G6PD axis inhibitors are promising pharmacological agents [270]. The pentose phosphate pathway (PPP) supports DNA replication and RNA production by regulating carbon flux between nucleic acid synthesis and lipogenesis, where key enzymes such as ribulose 5-phosphate isomerase (RPIA) and ribulose-5-phosphate-3-epimerase (RPE) are upregulated to maintain cell proliferation [271]. Studies have shown that S100A11 can enhance transketolase (TKT) synthesis and promote PPP [272]. In addition, the acidic tumor microenvironment can activate the YAP/MMP1 axis, promote the transition of cell metabolism to PPP, and promote tumor progression [273]. Elevated G6PD expression is also an important factor leading to erlotinib resistance [274]. Shukla et al. showed that mucin 1 (MUC1) and hypoxia-inducible factor 1α (HIF1α) increased deoxyCTP (dCTP) pools, thus inducing acquired resistance to gemcitabine through molecular competition [275]. ARNTL2-mediated cellular glycolysis was shown to increase sensitivity to erlotinib treatment through the activation of the PI3K/AKT signalling pathway [276]. Studies have also confirmed that the ERK/E2F1 pathway can cause gemcitabine resistance [277]. In amino acid metabolism, glutamine may contribute to gemcitabine resistance due to its role in controlling ROS and activating mTOR [269]. A nutrient-deficient environment may help activate the RNA-binding protein HuR, thereby upregulating isocitrate dehydrogenase 1 (IDH1) expression, enhancing antioxidant defence, and leading to chemotherapy resistance [278]. Increased glutamine uptake caused by MUC5AC overexpression can cause gemcitabine resistance [279]. The upregulation of FASN expression can induce endoplasmic reticulum stress and promote gemcitabine resistance by enhancing de novo lipid synthesis [92]. The inhibition of cholesterol synthesis may also enhance the effect of anticancer therapy [280], and the use of lipid rafts characterized by Cav-1-cholesterol may be a breakthrough therapeutic approach [281]. By targeting CRABP-II, lipid raft cholesterol accumulation can be reversed to overcome drug resistance [282]. CD36 expression is correlated with antiapoptotic protein expression and may protect PDAC cells from drug-induced cell death [283]. In addition, UBE2T can affect the remodelling of pyrimidine metabolism and confer gemcitabine resistance [284].

### Immunotherapy

Immunotherapy has gradually developed into a mature antitumour strategy, but its efficacy in PDAC is limited [285]. Some studies have attempted to predict the response to immunotherapy based on metabolic characteristics [286]. Metabolic disorders of the immune system in the TME are expected to improve the efficacy of metabolism-targeted anticancer strategies [287, 288].

Nucleotide metabolism provides genetic material and energy resources for immune system activation and proliferation [289]. Purine analogues, such as released extracellular ATP or adenosine, activate the purinergic and adenosine receptors of immune cells, thereby promoting or suppressing immune responses [290]. IFN triggers cell cycle arrest in the S phase, resulting in insufficient nucleotide pools and nucleoside efflux, and IFN combined with ATR inhibitors can induce lethal DNA damage and decrease nucleotide biosynthesis, thereby limiting the growth of PDAC [291].

Understanding the influence of metabolic regulation on the efficacy of immunotherapy is still in its infancy. Modulating cell metabolism via the autologous T cell adoptive transfer protocol can promote the generation of T cells with a memory phenotype and improve their antitumour effect [292, 293]. Engineering oncolytic viruses to express leptin can also enhance antitumour responses by increasing FAO and OXPHOS [294]. PD-1 blockade immunotherapy is a widely used therapy, but PDAC patients respond poorly. Targeting glycolysis impacts the accumulation of PD-1+ TILs in PDAC preclinical models [295]. Activating the AMPK and mTOR pathways related to mitochondrial metabolism is also expected to enhance the therapeutic effect of PD-1 blockers [296, 297].

Based on the aforementioned immune-metabolic crosstalk, new therapeutic targets have been investigated. For example, indoleamine 2,3-dioxygenase (IDO), which can degrade tryptophan and inhibit immune responses [298], has been explored in several clinical studies, which we will describe below.

### Other therapies

Other drug therapies are gradually being explored through emerging technologies [299]. The use of some nanomedicines can enhance oxidative stress [300]. Reduction-responsive nanoplatforms that can regulate lipid metabolism and polarize macrophages are available for the combined treatment of PDAC [301]. A novel dendrimer nanogel enhances chemoimmunotherapy through endoplasmic reticulum stress [302]. After degrading the ECM, nanoparticle transport can be enhanced, increasing the killing effect on PDAC [303, 304]. Studies have shown that selenoorganic compounds and dihydroartemisinin can induce ferroptosis in PDAC cells [305, 306]. Tailored ruthenium complexes can also affect OXPHOS and exert anticancer effects [307]. Although such investigations are still in the preclinical stage, they are providing new directions for the treatment of PDAC.

## Clinical trials targeting metabolism

Metabolic targeted therapy still has a long way to go before being applied as a routine treatment (Table 1). Some metabolic modulators, such as metformin [308] and statins [309], have been used to exert additional cancer-treatment effects. Metformin can affect the malignant behaviour of SMAD4-deficient PDAC cells by inhibiting HNF4G activity [310]; it can also destroy the dense matrix by inhibiting PSC activity, significantly improving the therapeutic efficacy of gemcitabine [311]. Several clinical trials of metformin for the treatment of PDAC, such as NCT02048384, NCT03889795 and NCT02201381, have been carried out. Through in vivo metabolism tracking, it was found that statins can inhibit the synthesis of coenzyme Q and that statins combined with MEK inhibitors can promote oxidative stress and apoptosis [312]. Based on these promising data, phase I and II clinical trials, such as NCT00944463, NCT00584012 and NCT04862260, were conducted. Irbesartan may reverse gemcitabine resistance by inhibiting iron metabolism [313]. In addition, drugs such as digoxin (NCT04141995), dapagliflozin (NCT04542291), omeprazole (NCT04930991), and even combinations of multiple metabolism-regulating drugs (NCT02201381) have been explored.

**Table 1:** Representative clinical trials targeting cancer metabolism in pancreatic cancer.

NCT identifier	Main target	Therapeutic agents	Intervention	Phase
NCT00096707	Glycolysis	2-deoxy-D-glucose (2-DG)	Monotherapy/Combination	I
NCT01835041	TCA cycle	CPI-613	Combination	I
NCT03504423	TCA cycle	CPI-613	Combination	III
NCT03965845	Glutamine	CB-839 (glutaminase inhibitor telaglenestat)	Combination	I
NCT04634539	Glutamine	L-glutamine	Combination	I
NCT01523808	Asparagine	GRASPA	Monotherapy	I
NCT02195180	Asparagine	GRASPA	Combination	II
NCT03665441	Asparagine	Eryaspase	Combination	III
NCT05034627	Asparagine/aspartate	Calaspargase pegol-mknl	Combination	I
NCT02077881	Tryptophan	Indoximod (IDO1 inhibitor)	Combination	I/II
NCT00739609	Tryptophan	1-methyl-D-tryptophan (IDO1 inhibitor)	Monotherapy	I
NCT03432676	Tryptophan	Epacadostat	Combination	II
NCT03006302	Tryptophan	Epacadostat and CRS-207	Combination	II
NCT03085914	Tryptophan	Epacadostat	Combination	I/II
NCT02101580	Arginine	ADI-PEG 20	Combination	I
NCT03435250	Methionine	AG-270	Monotherapy/Combination	I
NCT02223247	Lipid synthesis	TVB-2640 (FASN inhibitor)	Combination	I
NCT03829436	Lipid synthesis	TPST-1120 (antagonist of PPARα)	Combination	I
NCT03450018	Ferroptosis	SLC-0111 (CA IX inhibitor)	Combination	I/II
NCT02353026	Redox balance	Artesunate	Monotherapy	I
NCT01049880	Redox balance	Ascorbic Acid	Combination	I
NCT02514031	Redox balance	ARQ-761 (beta-lapachone)	Combination	I
NCT03825289	Autophagy	Choloroquine	Combination	II
NCT01777477	Autophagy	Choloroquine	Combination	I
NCT04132505	Autophagy	Choloroquine	Combination	I
NCT01019382	Dietary intervention	Omega-3 fatty acid	Combination	II
NCT01419483	Dietary intervention	Ketogenic diet	Combination	I
NCT02336087	Dietary intervention	Dietary supplements+ Metformin	Combination	I
NCT04930991	Metabolic Regulation	Omeprazole	Monotherapy	I
NCT04141995	Metabolic Regulation	Digoxin	Combination	II
NCT03889795	Metabolic Regulation	Simvastatin+ Digoxin+ Metformin	Combination	I
NCT02048384	Metabolic Regulation	Metformin	Combination	I
NCT04542291	Metabolic Regulation	Dapagliflozin	Monotherapy	I
NCT04862260	Metabolic Regulation	Evolocumab + Atorvastatin+ Ezetimibe	Combination	I
NCT00944463	Metabolic Regulation	Simvastatin	Combination	II
NCT00584012	Metabolic Regulation	Lovastatin	Combination	I
NCT02201381	Metabolic Regulation metabolism-regulating drugs	Metformin +Atorvastatin +Doxycycline +Mebendazole	Combination	III

Clinical trials have begun to explore treatments based on the glycolytic pathway. In the case of NCT00096707, 2-deoxy-D-glucose (2-DG) combined with docetaxel was used for advanced solid tumours and had tolerable adverse effects and certain clinical benefits [314]. The lipoic acid analogue CPI-613 was shown to selectively inhibit PDH activity and inhibit the growth of PDAC [315]. In NCT01835041 and NCT03504423, CPI-613 was combined with chemotherapy for the treatment of metastatic PDAC, yielding a good response rate [316].

Inducing metabolic crises through the antagonism of glutamine inhibitors, such as 6-diazo-5-oxo-L-norleucine (DON) and DRP-104, is a promising strategy in preclinical models [317]. NCT03965845 explored the efficacy of the glutaminase inhibitor telaglenastat (CB-839) in advanced or metastatic solid tumours. In NCT04634539, L-glutamine was combined with standard chemotherapy for advanced PDAC treatment. In recent years, in NCT01523808, NCT02195180, and NCT03665441, the combination of red blood cell-encapsulated asparaginase (such as GRASPA and eryaspase) with chemotherapy has been shown to improve the prognosis of patients, indicating its great potential as a second-line treatment [318]. Calaspargase pegol-mknl, which converts the amino acid L-asparagine into aspartic acid and ammonia, leading to cell death, was recently used in combination with cobimetinib in NCT05034627. The role of tryptophan and IDO1 in the immune microenvironment has recently been emphasized. In NCT00739609, NCT03432676, and NCT03006302, IDO1 inhibitors and PD-1 checkpoint inhibitors caused PDAC to respond to immunotherapy [319], and their efficacy in combination with chemotherapy has been further explored in clinical trials, such as NCT02077881 and NCT03085914. In addition, research on arginine (NCT02101580) and methionine (NCT03435250) has improved the understanding of targeting amino acid metabolism to treat PDAC.

Although lipid synthesis-related genes are overexpressed in a variety of cancers, relevant clinical trials are still exploratory. The application potential of the FASN inhibitor TVB-2640 and an antagonist of PPARα TPST-1120 were explored in NCT02223247 and NCT03829436, respectively. NCT03450018 was conducted to determine whether the CA IX inhibitor SLC-0111 exerts a synergistic anticancer effect by affecting ferroptosis.

There are also several drugs that may exert anticancer effects by affecting redox balance. For example, phase I clinical trials of artesunate and ascorbic acid have been conducted (NCT02353026 and NCT01049880, respectively). Combining β-lapachone (ARQ761) with a GLS1 inhibitor can induce excessive ROS production and selectively lead to PDAC cell death in preclinical models [320]. The potential of ARQ761 combined with gemcitabine/nab-paclitaxel treatment was explored in NCT02514031.

Autophagy plays an important role in the progression of PDAC, and the lysosomal inhibitor hydroxychloroquine may have synergistic effects with the MEK inhibitor trametinib. In this context, numerous clinical trials have been carried out (NCT01777477, NCT03825289, and NCT04132505).

Finally, there are some clinical trials based on dietary interventions, such as NCT01019382 and NCT01419483, but they are all in their early stages. In NCT02336087, a variety of dietary supplements were investigated, among which epigallocatechin-3 gallate (EGCG) may act by inhibiting FASN. However, these interventions are still far from clinical application.

## Conclusion and future perspective

**Fig. 2:**
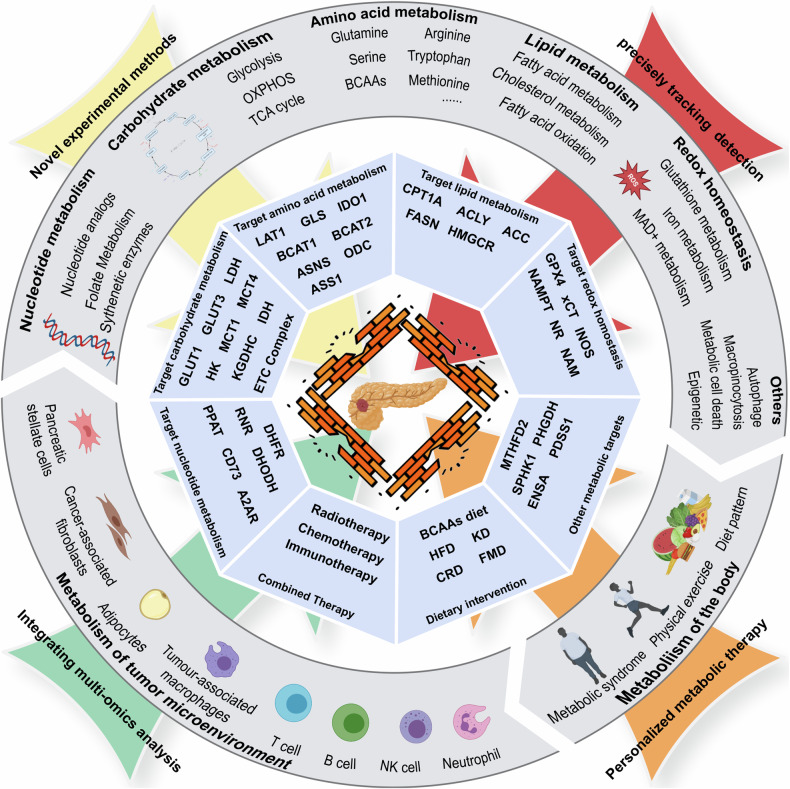
Overview of metabolic characteristics and therapy targets of pancreatic cancer. In addition to cellular metabolism such as nucleotide metabolism, carbohydrate metabolism, amino acid metabolism, lipid metabolism and oxidative homeostasis, complex tumor microenvironment metabolism and body metabolism (outer gray circles) together construct the metabolic characteristics of pancreatic cancer. With the development of novel experimental methods, precise tracking detection, integrated multi-omics analysis and personalized metabolic therapy (arrows), therapeutic or sensitization targets (blue regular octagons in the middle layer) have gradually been revealed, which is expected to break through the barriers to pancreatic cancer treatment (innermost), bringing new hope for the diagnosis and treatment of pancreatic cancer.

In recent years, the metabolic mechanism and translational application of PDAC treatment have become new and challenging research directions [321]. Both in vivo and in vitro experiments have revealed the metabolic dependence of PDAC [322, 323]. The use of stable isotopes has become a key method for exploring metabolic pathways in PDAC [324]. Advanced technology platforms such as organoids are also promoting advancements in the translation of metabolism-related research [325]. Many molecular subtyping methods based on metabolic characteristics are being developed for the treatment and evaluation of different types of PDAC patients [270]. Moreover, the development of single-cell sequencing and spatial omics has made it possible to characterize the metabolic crosstalk of different tissues at the cellular level [326]. Our understanding of the function of cellular metabolism in disease progression has progressed significantly (Fig. 2).

Given that most of the current understanding of how metabolism supports cell proliferation is based on studies in cancer cells, how metabolism shapes the interactions of different cells needs be explored in more complex ecosystems. This information is expected to help us gain a deeper understanding of the metabolic regulatory network, thereby overcoming the limitations of previous metabolic therapies and providing opportunities for tailor-made treatment plans for patients.

### Supplementary information

Supplemental Material
