# Supplementary material for: Emerging mechanisms and promising approaches in pancreatic cancer metabolism
Source: Cell Death Dis. 2024 Aug 1;15(8):553. doi: 10.1038/s41419-024-06930-0 (PMC11294586; doi:10.1038/s41419-024-06930-0)
Supplement: Supplementary file 1 — Supplemental Material [file 41419_2024_6930_MOESM1_ESM.docx]

**Text Box S1: Nucleotide metabolism**

Cancer cells must synthesize and utilize large amounts of energy and nucleotides to generate DNA and RNA, and purines and pyrimidines can be synthesized through the de novo pathway or the salvage pathway. Oncogenic KRAS has been shown to support pancreatic cancer by regulating nucleotide synthesis^1^. The earliest studies investigated limiting folate metabolism^2^, and dihydrofolate reductase (DHFR) inhibitors^3^, such as methotrexate, have been shown to work primarily through the consumption of folate cycle intermediates. Thymidylate synthase (TS) inhibitors, such as 5-fluorouracil (5-FU) and the folic acid analogue pemetrexed, can inhibit the de novo synthesis of thymidine and purine nucleotides^4^. In addition, the regulation of nucleotide biosynthesis is also controlled by negative feedback at the substrate level. Many purine and pyrimidine analogues, such as 6-mercaptopurine, 5-FU, gemcitabine, capecitabine, and fludarabine, are widely used in the clinical treatment of pancreatic cancer and other tumours^5, 6^.

The metabolic enzymes necessary for de novo nucleotide metabolism play important roles in maintaining nucleotide biosynthesis, and inhibiting these key enzymes is a promising therapeutic strategy. Ribonucleotide reductase (RNR) plays a key role in balancing dNTP levels, and inhibiting RNR with hydroxyurea has been approved by the United States Food and Drug Administration (FDA)^7^. DHODH is a key enzyme in the de novo pyrimidine pathway and is required for the synthesis of uridine monophosphate (UMP) from aspartate^8^. Uridine-derived ribose may provide fuel for treating pancreatic ductal adenocarcinoma (PDAC) under glucose deficiency^9^. Blocking DHODH can effectively inhibit the growth of various malignant tumours^10, 11^, and a recent study demonstrated that equilibrative nucleoside transporters 1 (ENT1) blockade by CNX-774 overcomes resistance to DHODH inhibition in pancreatic cancer^12^. Inosine monophosphate (IMP) is an intermediate in the de novo purine pathway that can be converted into AMP or GMP and has achieved promising results in preclinical experiments^13^. However, neither of the aforementioned treatments has received FDA approval to date.

There are several key enzymes involved in nucleotide metabolism, such as ecto-nucleoside triphosphate diphosphohydrolase-1 (ENTPD1, also known as CD39)^14^, ecto-5-nucleotidase (NT5E, also known as CD73)^15^, thymidine phosphorylase (TYMP)^16^, phosphoribosyl pyrophosphate aminotransferase (PPAT)^17^, inosine monophosphate dehydrogenases 1 and 2 (IMPDH1/2)^18^ and ENT1/2 ^19^, investigations of which continue to broaden our understanding of the role of these inhibitors in cancer. According to current basic research on pancreatic cancer, PPDPF can bind GTP and transfer GTP to SOS1 to promote the development of mutant KRAS-driven PDAC^20^. Dekhne et al. designed novel small-molecule pyrrolo[3,2-d] pyrimidine inhibitors targeting mitochondrial C1 metabolism at SHMT2, which generated significant in vivo antitumour efficacy^21^. Olou et al. reported that deoxyuridine could abrogate ROS-induced ER stress to promote cancer cell survival via the CDA-mediated reprogramming of pyrimidine metabolism^22^.

**Text Box S2: Oxidative phosphorylation (OXPHOS) in PDAC**

Oxidative phosphorylation (OXPHOS) is a key process connecting the tricarboxylic acid cycle (TCA) and the generation of adenosine triphosphate (ATP), and is the final biochemical pathway for producing ATP. Various tumor cell subpopulations have been found to be strongly dependent on the OXPHOS process, and KRAS mutant cells often show increased dependence on OXPHOS.

OXPHOS is mediated by five protein complexes (complex Ⅰ-Ⅴ) on the inner membrane of mitochondria. Mitochondria are important organelles that play irreplaceable roles in metabolic processes^23^, and their relationship with cancer is still being studied^24^. When PDAC cells survive the shutdown of oncogenic signalling, they are highly sensitive to oxidative phosphorylation inhibitors^25^. Mitochondrial fusion enhances mitophagy and proportionally induces tumour suppression^26^. Drugs based on mitochondrial metabolism have shown good therapeutic effects in early clinical trials^27^.

Tumor adaptation to aerobic glycolysis does not mean that OXPHOS is completely stopped^28^. By enhancing oxidative phosphorylation, it can promote the stem and immunoevasive properties of pancreatic cancer stem cells ^29^ and play a role in pancreatic cancer liver metastasis^30^. In chemotherapy-resistant tumors, OXPHOS is often upregulated^31^.

Therefore, many studies have explored the key enzymes in the oxidative phosphorylation process and specific participants in related pathways. The multimeric complex of the OXPHOS pathway is a target for small molecule inhibitors. Ubiquinol-cytochrome c reductase core protein I (UQCRC1), as a key component of mitochondrial complex III, can lead to increased OXPHOS and ATP production, and has a pro-carcinogenic effect^32^.

Knockout of BCAT2 inhibits the malignant progression of pancreatic cancer by regulating the catabolism of branched-chain amino acids and reducing the oxidative phosphorylation ability of pancreatic cancer cells^33^. Methionine sulfoxide reductase A (MSRA) can act as a reversible redox switch and play the role of a tumor suppressor^34^. Studies have found that the NNMT-DNMT1 axis can maintain cancer cell OXPHOS dependence^35^. Recent studies have found that SETD2 deletion mutations can enhance OXPHOS dependent on fatty acid metabolism and promote lipid-loaded tumor-associated fibroblasts (CAFs) to promote pancreatic cancer progression, providing new ideas for treatment^36^.

Based on the above ideas, antidiabetic biguanides and antiparasitic drugs have been repurposed to interfere with OXPHOS to assist cancer treatment. Metformin targets mitochondrial metabolism at clinical doses^37^. Phenformin has stronger potency and lipid solubility than metformin, but there is a risk of systemic lactic acidosis^38^.

Traditional OXPHOS inhibitors are usually too toxic to be used in clinical cancer treatment, and inhibitors with preferential toxicity to cancer cells need to be developed. Nouri Neamati et al. discovered OXPHOS inhibitors DX3-234 and DX3-213B based on pancreatic cancer cell lines^39, 40^. There are also some new OXPHOS-based nano-drugs, which we will list later.

**Text Box S3: Amino acid metabolism**

Serine plays an important role in one-carbon metabolism to support cancer cell proliferation and growth^41, 42^. Cells take up exogenous serine through the serine transporter ASCT1 or synthesize serine de novo through the serine synthesis pathway. Meanwhile, KRAS-driven PDAC mouse models were less responsive to a depletion of serine and glycine^43^. Macropinocytosis and autophagy are nonclassical sources of serine uptake^44^. Multiple mechanisms activate the expression of the ASCT1 and SSP genes in cancer^45^. Phgdh inhibition and dietary serine restriction have been shown to have antitumour effects on mouse cancer models^46^, but tumour therapy based on serine metabolism still faces considerable challenges^47^. Type II transmembrane serine proteases (TTSPs) are overexpressed in a variety of tumours and are potential new markers^48^. In genetically engineered mouse models, liver kinase B1 (LKB1) deficiency sensitizes tumors to serine biosynthesis, suggesting a potential therapeutic vulnerability^49^.

Indoleamine-2,3-dioxygenase 1 (IDO1) and tryptophan 2,3-dioxygenase (TDO) can catalyse the conversion of tryptophan to kynurenine, which can regulate the invasion and metastasis of PDAC^50^, and kynurenine supplementation can inhibit tumours caused by IDO1/TDO knockdown through the aryl hydrocarbon receptor (AHR) pathway^51^.

Methionine consumption exceeds methionine synthesis in cancer cells^52^, and the dependence of cancer cells on exogenous methionine is called methionine dependence or the Hoffman effect^53^. Methionine adenosyltransferase (MAT) synthesizes S-adenosylmethionine (SAM) from methionine, providing a methyl group for methylation, and is a potential therapeutic target^54^. Methionine obtained from the diet may contribute to diet-affected tumour-specific metabolic vulnerability through tumour cell metabolism^55^. However, some cohort studies have shown that the serum methionine concentration is inversely related to the risk of pancreatic cancer^56^.

Leucine and arginine are the most important amino acid regulators of the mTOR pathway^57, 58^. Leucine can activate the mTOR signalling pathway through SLC38A9 and promote the proliferation of pancreatic cancer cells^59^. The addition of L-citrulline can rescue autophagy-dependent cell death caused by arginine deprivation in PDAC cells expressing ASS1^60^. Recent studies have shown that RBM39 senses arginine, leading to metabolic reprogramming and promoting tumour development^61^. Arginine deprivation is a potential strategy for PDAC treatment^62^.

The role of branched-chain amino acids (BCAAs) in cancer has gradually been revealed^63^. The stabilization of branched-chain amino acid transaminase 2 (BCAT2) enhances PDAC proliferation^64^, and the addition of branched-chain α-keto acid (BCKA) and nucleobases rescues the growth of organoids suppressed by BCAT2 inhibitors^33^. The knockdown of branched-chain ketoacid dehydrogenase kinase (BCKDK) can inhibit pancreatic cancer growth^65^. However, adding different concentrations of BCAAs to the culture medium failed to promote the proliferation and migration of PDAC cells^66^. This may be attributed to reduced BCAA uptake and utilization in KRAS-driven pancreatic cancer, rather than the requirement of BCAAs for growth support^67^. Therefore, BCAAs may play different roles in different stages of pancreatic cancer or in different microenvironments, but this requires further research.

**Text Box S4: Dietary intervention**

A high-fat diet (HFD) is the most well-known factor that promotes pancreatic cancer^68, 69^. A HFD can exacerbate lipid metabolic reprogramming, thereby driving cancer development^70^. Continuous inflammatory stimulation, autophagy dysregulation, and a reduction in DNA damage may be intrinsic mechanisms of PDAC tumorigenesis and invasion^71, 72, 73^. A calorie-restricted diet (CRD) reduces daily caloric intake by 20%–40% while ensuring essential nutrients for the body^74^; this can inhibit cancer by activating antitumour immunity and reducing polyunsaturated fatty acids (PUFAs)^75, 76^. A fasting-mimicking diet (FMD) is based on a CRD and includes a very low-calorie dieting period that generally lasts 4–5 days^77^; it can achieve a tumour suppressive effect by reducing the levels of circulating insulin-like growth factor 1, leptin and insulin. It can also increase the resistance of normal cells to chemotherapy^78, 79^. A ketogenic diet (KD) is a dietary regimen that mimics the fasting state and induces the production of ketone bodies. It has received widespread attention in recent years^80^, with results indicating that it is possible to explore and manipulate amino acid intake to activate immune responses and control tumour progression^81, 82^, and clinical trials have been carried out with patients with colorectal cancer^83^. In pancreatic cancer, a ketogenic diet combined with gemcitabine improved survival in mice^84^. The intake of a diet rich in branched-chain amino acids, which are associated with an increased risk of PDAC^85^, may stabilize BCAT2 by upregulating USP1 expression^86^.

**Reference**

1. Santana-Codina N, Roeth AA, Zhang Y, Yang A, Mashadova O, Asara JM*, et al.* Oncogenic KRAS supports pancreatic cancer through regulation of nucleotide synthesis. *Nat Commun* 2018, **9**(1)**:** 4945.

2. Sun J, Wei Q, Zhou Y, Wang J, Liu Q, Xu H. A systematic analysis of FDA-approved anticancer drugs. *BMC Syst Biol* 2017, **11**(Suppl 5)**:** 87.

3. Waltham MC, Holland JW, Robinson SC, Winzor DJ, Nixon PF. Direct experimental evidence for competitive inhibition of dihydrofolate reductase by methotrexate. *Biochem Pharmacol* 1988, **37**(3)**:** 535-539.

4. Adjei AA. Pharmacology and mechanism of action of pemetrexed. *Clin Lung Cancer* 2004, **5 Suppl 2:** S51-55.

5. Shelton J, Lu X, Hollenbaugh JA, Cho JH, Amblard F, Schinazi RF. Metabolism, Biochemical Actions, and Chemical Synthesis of Anticancer Nucleosides, Nucleotides, and Base Analogs. *Chem Rev* 2016, **116**(23)**:** 14379-14455.

6. Parker WB. Enzymology of purine and pyrimidine antimetabolites used in the treatment of cancer. *Chem Rev* 2009, **109**(7)**:** 2880-2893.

7. Huff SE, Winter JM, Dealwis CG. Inhibitors of the Cancer Target Ribonucleotide Reductase, Past and Present. *Biomolecules* 2022, **12**(6).

8. Koundinya M, Sudhalter J, Courjaud A, Lionne B, Touyer G, Bonnet L*, et al.* Dependence on the Pyrimidine Biosynthetic Enzyme DHODH Is a Synthetic Lethal Vulnerability in Mutant KRAS-Driven Cancers. *Cell Chem Biol* 2018, **25**(6)**:** 705-717 e711.

9. Nwosu ZC, Ward MH, Sajjakulnukit P, Poudel P, Ragulan C, Kasperek S*, et al.* Uridine-derived ribose fuels glucose-restricted pancreatic cancer. *Nature* 2023, **618**(7963)**:** 151-158.

10. Shi DD, Savani MR, Levitt MM, Wang AC, Endress JE, Bird CE*, et al.* De novo pyrimidine synthesis is a targetable vulnerability in IDH mutant glioma. *Cancer Cell* 2022, **40**(9)**:** 939-956 e916.

11. Jin R, Liu B, Liu X, Fan Y, Peng W, Huang C*, et al.* Leflunomide Suppresses the Growth of LKB1-Inactivated Tumors in the Immune-Competent Host and Attenuates Distant Cancer Metastasis. *Mol Cancer Ther* 2021, **20**(2)**:** 274-283.

12. Mullen NJ, Thakur R, Shukla SK, Chaika NV, Kollala SS, Wang D*, et al.* ENT1 blockade by CNX-774 overcomes resistance to DHODH inhibition in pancreatic cancer. *Cancer Lett* 2023, **552:** 215981.

13. Naffouje R, Grover P, Yu H, Sendilnathan A, Wolfe K, Majd N*, et al.* Anti-Tumor Potential of IMP Dehydrogenase Inhibitors: A Century-Long Story. *Cancers (Basel)* 2019, **11**(9).

14. Cai XY, Wang XF, Li J, Dong JN, Liu JQ, Li NP*, et al.* High expression of CD39 in gastric cancer reduces patient outcome following radical resection. *Oncol Lett* 2016, **12**(5)**:** 4080-4086.

15. King RJ, Shukla SK, He C, Vernucci E, Thakur R, Attri KS*, et al.* CD73 induces GM-CSF/MDSC-mediated suppression of T cells to accelerate pancreatic cancer pathogenesis. *Oncogene* 2022, **41**(7)**:** 971-982.

16. Liang K, Abt ER, Le TM, Cho A, Dann AM, Cui J*, et al.* STING-driven interferon signaling triggers metabolic alterations in pancreas cancer cells visualized by [(18)F]FLT PET imaging. *Proc Natl Acad Sci U S A* 2021, **118**(36).

17. Fridman A, Saha A, Chan A, Casteel DE, Pilz RB, Boss GR. Cell cycle regulation of purine synthesis by phosphoribosyl pyrophosphate and inorganic phosphate. *Biochem J* 2013, **454**(1)**:** 91-99.

18. Ni C, Liu W, Zheng K, Guo S, Song B, Jing W*, et al.* PI3K/ c-Myc/AFF4 axis promotes pancreatic tumorigenesis through fueling nucleotide metabolism. *Int J Biol Sci* 2023, **19**(6)**:** 1968-1982.

19. Abt ER, Rosser EW, Durst MA, Lok V, Poddar S, Le TM*, et al.* Metabolic Modifier Screen Reveals Secondary Targets of Protein Kinase Inhibitors within Nucleotide Metabolism. *Cell Chem Biol* 2020, **27**(2)**:** 197-205 e196.

20. Ni QZ, Zhu B, Ji Y, Zheng QW, Liang X, Ma N*, et al.* PPDPF Promotes the Development of Mutant KRAS-Driven Pancreatic Ductal Adenocarcinoma by Regulating the GEF Activity of SOS1. *Adv Sci (Weinh)* 2023, **10**(2)**:** e2202448.

21. Dekhne AS, Shah K, Ducker GS, Katinas JM, Wong-Roushar J, Nayeen MJ*, et al.* Novel Pyrrolo[3,2-d]pyrimidine Compounds Target Mitochondrial and Cytosolic One-carbon Metabolism with Broad-spectrum Antitumor Efficacy. *Mol Cancer Ther* 2019, **18**(10)**:** 1787-1799.

22. Olou AA, King RJ, Yu F, Singh PK. MUC1 oncoprotein mitigates ER stress via CDA-mediated reprogramming of pyrimidine metabolism. *Oncogene* 2020, **39**(16)**:** 3381-3395.

23. Spinelli JB, Haigis MC. The multifaceted contributions of mitochondria to cellular metabolism. *Nat Cell Biol* 2018, **20**(7)**:** 745-754.

24. Kopinski PK, Singh LN, Zhang S, Lott MT, Wallace DC. Mitochondrial DNA variation and cancer. *Nat Rev Cancer* 2021, **21**(7)**:** 431-445.

25. Viale A, Pettazzoni P, Lyssiotis CA, Ying H, Sanchez N, Marchesini M*, et al.* Oncogene ablation-resistant pancreatic cancer cells depend on mitochondrial function. *Nature* 2014, **514**(7524)**:** 628-632.

26. Yu M, Nguyen ND, Huang Y, Lin D, Fujimoto TN, Molkentine JM*, et al.* Mitochondrial fusion exploits a therapeutic vulnerability of pancreatic cancer. *JCI Insight* 2019, **5**(16).

27. Philip PA, Buyse ME, Alistar AT, Rocha Lima CM, Luther S, Pardee TS*, et al.* A Phase III open-label trial to evaluate efficacy and safety of CPI-613 plus modified FOLFIRINOX (mFFX) versus FOLFIRINOX (FFX) in patients with metastatic adenocarcinoma of the pancreas. *Future Oncol* 2019, **15**(28)**:** 3189-3196.

28. Greene J, Segaran A, Lord S. Targeting OXPHOS and the electron transport chain in cancer; Molecular and therapeutic implications. *Semin Cancer Biol* 2022, **86**(Pt 2)**:** 851-859.

29. Valle S, Alcala S, Martin-Hijano L, Cabezas-Sainz P, Navarro D, Munoz ER*, et al.* Exploiting oxidative phosphorylation to promote the stem and immunoevasive properties of pancreatic cancer stem cells. *Nat Commun* 2020, **11**(1)**:** 5265.

30. Fabian A, Stegner S, Miarka L, Zimmermann J, Lenk L, Rahn S*, et al.* Metastasis of pancreatic cancer: An uninflamed liver micromilieu controls cell growth and cancer stem cell properties by oxidative phosphorylation in pancreatic ductal epithelial cells. *Cancer Lett* 2019, **453:** 95-106.

31. Uslu C, Kapan E, Lyakhovich A. Cancer resistance and metastasis are maintained through oxidative phosphorylation. *Cancer Lett* 2024, **587:** 216705.

32. Wang Q, Li M, Gan Y, Jiang S, Qiao J, Zhang W*, et al.* Mitochondrial Protein UQCRC1 is Oncogenic and a Potential Therapeutic Target for Pancreatic Cancer. *Theranostics* 2020, **10**(5)**:** 2141-2157.

33. Li JT, Yin M, Wang D, Wang J, Lei MZ, Zhang Y*, et al.* BCAT2-mediated BCAA catabolism is critical for development of pancreatic ductal adenocarcinoma. *Nat Cell Biol* 2020, **22**(2)**:** 167-174.

34. He D, Feng H, Sundberg B, Yang J, Powers J, Christian AH*, et al.* Methionine oxidation activates pyruvate kinase M2 to promote pancreatic cancer metastasis. *Mol Cell* 2022, **82**(16)**:** 3045-3060 e3011.

35. Wu C, Liu Y, Liu W, Zou T, Lu S, Zhu C*, et al.* NNMT-DNMT1 Axis is Essential for Maintaining Cancer Cell Sensitivity to Oxidative Phosphorylation Inhibition. *Adv Sci (Weinh)* 2022, **10**(1)**:** e2202642.

36. Niu N, Shen X, Wang Z, Chen Y, Weng Y, Yu F*, et al.* Tumor cell-intrinsic epigenetic dysregulation shapes cancer-associated fibroblasts heterogeneity to metabolically support pancreatic cancer. *Cancer Cell* 2024, **42**(5)**:** 869-884 e869.

37. Lord SR, Cheng WC, Liu D, Gaude E, Haider S, Metcalf T*, et al.* Integrated Pharmacodynamic Analysis Identifies Two Metabolic Adaption Pathways to Metformin in Breast Cancer. *Cell Metab* 2018, **28**(5)**:** 679-688 e674.

38. Vial G, Detaille D, Guigas B. Role of Mitochondria in the Mechanism(s) of Action of Metformin. *Front Endocrinol (Lausanne)* 2019, **10:** 294.

39. Xue D, Xu Y, Kyani A, Roy J, Dai L, Sun D*, et al.* Discovery and Lead Optimization of Benzene-1,4-disulfonamides as Oxidative Phosphorylation Inhibitors. *J Med Chem* 2022, **65**(1)**:** 343-368.

40. Xue D, Xu Y, Kyani A, Roy J, Dai L, Sun D*, et al.* Multiparameter Optimization of Oxidative Phosphorylation Inhibitors for the Treatment of Pancreatic Cancer. *J Med Chem* 2022, **65**(4)**:** 3404-3419.

41. Geeraerts SL, Heylen E, De Keersmaecker K, Kampen KR. The ins and outs of serine and glycine metabolism in cancer. *Nat Metab* 2021, **3**(2)**:** 131-141.

42. Ducker GS, Rabinowitz JD. One-Carbon Metabolism in Health and Disease. *Cell Metab* 2017, **25**(1)**:** 27-42.

43. Maddocks ODK, Athineos D, Cheung EC, Lee P, Zhang T, van den Broek NJF*, et al.* Modulating the therapeutic response of tumours to dietary serine and glycine starvation. *Nature* 2017, **544**(7650)**:** 372-376.

44. Kamphorst JJ, Nofal M, Commisso C, Hackett SR, Lu W, Grabocka E*, et al.* Human pancreatic cancer tumors are nutrient poor and tumor cells actively scavenge extracellular protein. *Cancer Res* 2015, **75**(3)**:** 544-553.

45. Tajan M, Hennequart M, Cheung EC, Zani F, Hock AK, Legrave N*, et al.* Serine synthesis pathway inhibition cooperates with dietary serine and glycine limitation for cancer therapy. *Nat Commun* 2021, **12**(1)**:** 366.

46. Muthusamy T, Cordes T, Handzlik MK, You L, Lim EW, Gengatharan J*, et al.* Serine restriction alters sphingolipid diversity to constrain tumour growth. *Nature* 2020, **586**(7831)**:** 790-795.

47. Rossi M, Altea-Manzano P, Demicco M, Doglioni G, Bornes L, Fukano M*, et al.* PHGDH heterogeneity potentiates cancer cell dissemination and metastasis. *Nature* 2022, **605**(7911)**:** 747-753.

48. Kim S. TMPRSS4, a type II transmembrane serine protease, as a potential therapeutic target in cancer. *Exp Mol Med* 2023, **55**(4)**:** 716-724.

49. Kottakis F, Nicolay BN, Roumane A, Karnik R, Gu H, Nagle JM*, et al.* LKB1 loss links serine metabolism to DNA methylation and tumorigenesis. *Nature* 2016, **539**(7629)**:** 390-395.

50. Liang H, Li T, Fang X, Xing Z, Zhang S, Shi L*, et al.* IDO1/TDO dual inhibitor RY103 targets Kyn-AhR pathway and exhibits preclinical efficacy on pancreatic cancer. *Cancer Lett* 2021, **522:** 32-43.

51. Wang L, Tang W, Yang S, He P, Wang J, Gaedcke J*, et al.* NO(*) /RUNX3/kynurenine metabolic signaling enhances disease aggressiveness in pancreatic cancer. *Int J Cancer* 2020, **146**(11)**:** 3160-3169.

52. Wang Z, Yip LY, Lee JHJ, Wu Z, Chew HY, Chong PKW*, et al.* Methionine is a metabolic dependency of tumor-initiating cells. *Nat Med* 2019, **25**(5)**:** 825-837.

53. Kaiser P. Methionine Dependence of Cancer. *Biomolecules* 2020, **10**(4).

54. Yang PW, Jiao JY, Chen Z, Zhu XY, Cheng CS. Keep a watchful eye on methionine adenosyltransferases, novel therapeutic opportunities for hepatobiliary and pancreatic tumours. *Biochim Biophys Acta Rev Cancer* 2022, **1877**(5)**:** 188793.

55. Sanderson SM, Gao X, Dai Z, Locasale JW. Methionine metabolism in health and cancer: a nexus of diet and precision medicine. *Nat Rev Cancer* 2019, **19**(11)**:** 625-637.

56. Huang JY, Luu HN, Butler LM, Midttun O, Ulvik A, Wang R*, et al.* A prospective evaluation of serum methionine-related metabolites in relation to pancreatic cancer risk in two prospective cohort studies. *Int J Cancer* 2020, **147**(7)**:** 1917-1927.

57. Liu KA, Lashinger LM, Rasmussen AJ, Hursting SD. Leucine supplementation differentially enhances pancreatic cancer growth in lean and overweight mice. *Cancer Metab* 2014, **2**(1)**:** 6.

58. Chen CL, Hsu SC, Ann DK, Yen Y, Kung HJ. Arginine Signaling and Cancer Metabolism. *Cancers (Basel)* 2021, **13**(14).

59. Wyant GA, Abu-Remaileh M, Wolfson RL, Chen WW, Freinkman E, Danai LV*, et al.* mTORC1 Activator SLC38A9 Is Required to Efflux Essential Amino Acids from Lysosomes and Use Protein as a Nutrient. *Cell* 2017, **171**(3)**:** 642-654 e612.

60. Khalil N, Abi-Habib RJ. [HuArgI (co)-PEG5000]-induced arginine deprivation leads to autophagy dependent cell death in pancreatic cancer cells. *Invest New Drugs* 2020, **38**(5)**:** 1236-1246.

61. Mossmann D, Muller C, Park S, Ryback B, Colombi M, Ritter N*, et al.* Arginine reprograms metabolism in liver cancer via RBM39. *Cell* 2023, **186**(23)**:** 5068-5083 e5023.

62. Yang JS, Wang CC, Qiu JD, Ren B, You L. Arginine metabolism: a potential target in pancreatic cancer therapy. *Chin Med J (Engl)* 2020, **134**(1)**:** 28-37.

63. Sivanand S, Vander Heiden MG. Emerging Roles for Branched-Chain Amino Acid Metabolism in Cancer. *Cancer Cell* 2020, **37**(2)**:** 147-156.

64. Lei MZ, Li XX, Zhang Y, Li JT, Zhang F, Wang YP*, et al.* Acetylation promotes BCAT2 degradation to suppress BCAA catabolism and pancreatic cancer growth. *Signal Transduct Target Ther* 2020, **5**(1)**:** 70.

65. Lee JH, Cho YR, Kim JH, Kim J, Nam HY, Kim SW*, et al.* Branched-chain amino acids sustain pancreatic cancer growth by regulating lipid metabolism. *Exp Mol Med* 2019, **51**(11)**:** 1-11.

66. Jiang W, Qiao L, Han Y, Zhang A, An H, Xiao J*, et al.* Pancreatic stellate cells regulate branched-chain amino acid metabolism in pancreatic cancer. *Ann Transl Med* 2021, **9**(5)**:** 417.

67. Mayers JR, Torrence ME, Danai LV, Papagiannakopoulos T, Davidson SM, Bauer MR*, et al.* Tissue of origin dictates branched-chain amino acid metabolism in mutant Kras-driven cancers. *Science* 2016, **353**(6304)**:** 1161-1165.

68. Chang HH, Moro A, Takakura K, Su HY, Mo A, Nakanishi M*, et al.* Incidence of pancreatic cancer is dramatically increased by a high fat, high calorie diet in KrasG12D mice. *PLoS One* 2017, **12**(9)**:** e0184455.

69. Liu B, Wang F, Chen L, Xin Y, Liu L, Wu D*, et al.* Effects of High-Fat Diet on Carcinogen-Induced Pancreatic Cancer and Intestinal Microbiota in C57BL/6 Wild-Type Mice. *Pancreas* 2021, **50**(4)**:** 564-570.

70. Zhang R, Peng X, Du JX, Boohaker R, Estevao IL, Grajeda BI*, et al.* Oncogenic KRASG12D Reprograms Lipid Metabolism by Upregulating SLC25A1 to Drive Pancreatic Tumorigenesis. *Cancer Res* 2023, **83**(22)**:** 3739-3752.

71. Guo X, Li J, Tang R, Zhang G, Zeng H, Wood RJ*, et al.* High Fat Diet Alters Gut Microbiota and the Expression of Paneth Cell-Antimicrobial Peptides Preceding Changes of Circulating Inflammatory Cytokines. *Mediators Inflamm* 2017, **2017:** 9474896.

72. Torres C, Mancinelli G, Cordoba-Chacon J, Viswakarma N, Castellanos K, Grimaldo S*, et al.* p110gamma deficiency protects against pancreatic carcinogenesis yet predisposes to diet-induced hepatotoxicity. *Proc Natl Acad Sci U S A* 2019, **116**(29)**:** 14724-14733.

73. Garcia DI, Hurst KE, Bradshaw A, Janakiraman H, Wang C, Camp ER. High-Fat Diet Drives an Aggressive Pancreatic Cancer Phenotype. *J Surg Res* 2021, **264:** 163-172.

74. Ibrahim EM, Al-Foheidi MH, Al-Mansour MM. Energy and caloric restriction, and fasting and cancer: a narrative review. *Support Care Cancer* 2021, **29**(5)**:** 2299-2304.

75. Lien EC, Westermark AM, Zhang Y, Yuan C, Li Z, Lau AN*, et al.* Low glycaemic diets alter lipid metabolism to influence tumour growth. *Nature* 2021, **599**(7884)**:** 302-307.

76. Pomatto-Watson LCD, Bodogai M, Bosompra O, Kato J, Wong S, Carpenter M*, et al.* Daily caloric restriction limits tumor growth more effectively than caloric cycling regardless of dietary composition. *Nat Commun* 2021, **12**(1)**:** 6201.

77. Sofi F. FASTING-MIMICKING DIET a clarion call for human nutrition research or an additional swan song for a commercial diet? *Int J Food Sci Nutr* 2020, **71**(8)**:** 921-928.

78. Caffa I, Spagnolo V, Vernieri C, Valdemarin F, Becherini P, Wei M*, et al.* Fasting-mimicking diet and hormone therapy induce breast cancer regression. *Nature* 2020, **583**(7817)**:** 620-624.

79. Nencioni A, Caffa I, Cortellino S, Longo VD. Fasting and cancer: molecular mechanisms and clinical application. *Nat Rev Cancer* 2018, **18**(11)**:** 707-719.

80. Zhu H, Bi D, Zhang Y, Kong C, Du J, Wu X*, et al.* Ketogenic diet for human diseases: the underlying mechanisms and potential for clinical implementations. *Signal Transduct Target Ther* 2022, **7**(1)**:** 11.

81. Dai X, Bu X, Gao Y, Guo J, Hu J, Jiang C*, et al.* Energy status dictates PD-L1 protein abundance and anti-tumor immunity to enable checkpoint blockade. *Mol Cell* 2021, **81**(11)**:** 2317-2331 e2316.

82. Tajan M, Vousden KH. Dietary Approaches to Cancer Therapy. *Cancer Cell* 2020, **37**(6)**:** 767-785.

83. Dmitrieva-Posocco O, Wong AC, Lundgren P, Golos AM, Descamps HC, Dohnalova L*, et al.* beta-Hydroxybutyrate suppresses colorectal cancer. *Nature* 2022, **605**(7908)**:** 160-165.

84. Cortez NE, Rodriguez Lanzi C, Hong BV, Xu J, Wang F, Chen S*, et al.* A ketogenic diet in combination with gemcitabine increases survival in pancreatic cancer KPC mice. *Cancer Res Commun* 2022, **2**(9)**:** 951-965.

85. Rossi M, Turati F, Strikoudi P, Ferraroni M, Parpinel M, Serraino D*, et al.* Dietary intake of branched-chain amino acids and pancreatic cancer risk in a case-control study from Italy. *Br J Nutr* 2022**:** 1-19.

86. Li JT, Li KY, Su Y, Shen Y, Lei MZ, Zhang F*, et al.* Diet high in branched-chain amino acid promotes PDAC development by USP1-mediated BCAT2 stabilization. *Natl Sci Rev* 2022, **9**(5)**:** nwab212.
